# Novel Interventions and Assessments Using Patient Portals in Adolescent Research: Confidential Survey Study

**DOI:** 10.2196/jmir.8340

**Published:** 2018-03-21

**Authors:** Lindsay A Thompson, Rebeccah Mercado, Thomas Martinko, Ratna Acharya

**Affiliations:** ^1^ Department of Pediatrics University of Florida Gainesville, FL United States; ^2^ Department of Health Outcomes and Biomedical Informatics University of Florida Gainesville, FL United States

**Keywords:** adolescent health services, preventive health services, health information technology

## Abstract

**Background:**

While adolescents can receive confidential health care without parental or guardian notification, they are rarely asked about their experiences and opinions regarding their care because participation in research often requires parental consent. Anonymous research with adolescents via confidential patient portals may ameliorate this research gap.

**Objective:**

Because use of a confidential online adolescent patient portal is high at our academic institution, we hypothesized that adolescents would also respond to survey-based research via the portal, especially if asked anonymously and without parental consent. We used a clinical scenario of needing to better understanding adolescent and young adults’ views about their health and health care, including information on a long-acting reversible contraceptive (LARC) to test if and how they will use a portal for research.

**Methods:**

Upon receiving Institutional Review Board approval, we sent 2 portal-based surveys about confidential services to 2 groups of females, ages 14 to 25 years, who had attended an adolescent clinic in the past 3 years. This clinic mostly serves Medicaid recipients (80%) and is racially and ethnically diverse with half of patients identifying as African American and roughly 10% Hispanic. The control group was a random sample of female patients who never received a LARC (n=150) and the intervention group included all female adolescents who had received a LARC from the same clinic (n=107). This second sample was manually cross-checked to confirm they had an office visit for this reason. Consenting for themselves, the control group received an email through the patient portal with a link and a request to perform an assessment. The survey for the control group included items assessing health literacy and health communication preferences. The survey for the intervention group included health literacy items as well as items to assess their opinions and perceptions regarding LARCs. We tracked click-through rates and opened messages; each participant received 4 reminders.

**Results:**

While only 3 participants fully completed either survey, email read rates (29/107 [27.1%] of LARC recipients and 39/150 [26.0%] of controls) were encouraging. Additionally, of those who opened the messages, almost twice as many of the LARC recipients (10/107 [9.3%]) read through the entire survey, while less than half read the entire survey as compared to those who received the survey asking about health literacy and health care preferences (6/150 [4.0%]).

**Conclusions:**

The methodology of using adolescent portals for online surveys provides a new avenue for research even though the study did not yield sufficient participation to understand these adolescents’ preferences. Future studies need to test if a different survey topic would engage adolescents or if other methods like text-based reminders would improve participation.

## Introduction

Adolescent health research often is overlooked or delayed due to the difficulties of maintaining appropriate confidentiality and privacy [1]. Adolescents can receive confidential health care covering family planning and sexually transmitted disease management without parental or guardian notification, but asking them about their experiences and opinions regarding that same care often requires parental consent. Because this ironic process risks breaking confidentiality, many research questions remain unanswered.

Research advances need to include developing novel and ethical methods of asking questions directly to adolescents. These methods should use caution and follow recommended observational research guidelines such as those created by Ruiz-Canela et al [1] that provide a decision tree to guide researchers, institutional review boards, and ethics committees on how to appropriately enroll adolescents in observational research studies. These authors support confidential research with adolescents without parental consent when 2 conditions are met: when risk is minimal and when there are “specific circumstances that might contribute to vulnerability” [1]. According to the Society for Adolescent Health and Medicine, confidential or anonymous survey research should be considered low risk in the adolescent population as with adults, specifically since deferring parental consent will avoid biasing results [2]. Requiring parental consent on surveys discussing protected information with adolescents potentially strains the parental relationship [2]. Additionally, breaches could break state laws that almost universally allow adolescents to seek family planning interventions without parental approval. For example, a study that required parental consent to ask adolescents about birth control methods would break legal confidentiality. Waivers of parental consent become logical and legal.

Consequently, researchers and adolescent specialists have urged development of alternative methods and locations for adolescent research, particularly survey research, to protect adolescent confidentiality. To date, these have primarily included school- and community-based studies [2]. To our knowledge, online methods of research, specifically confidential patient portals, have yet to be studied. Adolescent portals have become increasingly popular for direct and confidential doctor-patient communication, especially those that maximize confidentiality and aim to educate adolescents on how to access and advocate for their own health needs [3]. Via a private, well-used online patient portal tailored to adolescents’ needs [4], we aimed to measure if and how adolescents use their patient portal to consider research participation. By anonymously seeking opinions without parental consent, we sought a research mechanism by which we could solicit adolescents’ knowledge and opinions about their health and health care they received, especially if they received a long-acting reversible contraceptive (LARC) whose insertion is protected by state confidentiality and family planning laws.

## Methods

### Study Population

We received institutional review board approval for this study to recruit young adults and adolescents via a waiver of parental consent. The target population was females aged 14 to 25 years who attended the University of Florida Adolescent Clinic between April 1, 2013, and March 31, 2016. This clinic mostly serves Medicaid recipients (80%) and is diverse, with patients evenly divided between African American and white adolescents and roughly 10% Hispanic adolescents.

### Online Patient Portal

In brief, this private adolescent portal was specifically designed for confidential communication between provider and adolescent and does not include parental access unless the adolescent specifically desires it [3,4]. It is widely used in this health care system, with over 60% of adolescents having an activated online portal [3].

### Survey Development

We designed 2 similar surveys for implementation that included demographics, the Newest Vital Sign [5], the Single Item Literacy Screener [6], and the Health-Care Self-Determination Theory Questionnaire [7]. For those who had received LARCs in our clinic, we adapted published questions on youth knowledge, experiences, and attitudes about LARC (personal communication with J Peipert, MD, October, 2015) [8,9]. None of the questions required an answer to proceed. We pilot-tested the survey with 16 college students aged 18 to 25 years to ensure the questions were generally understandable. Recommended revisions only changed about 5% of the questions, so we did not perform additional iterations of the evaluation. Both surveys had an 8th grade reading level and would take no longer than 20 minutes to complete.

### Identification of Participants

We identified 2 patient populations via the university’s online database. First, a random sample of female patients aged 14 to 25 years who had attended the Adolescent Clinic but never received a LARC was identified and generated by the electronic database (n=150). Second, we requested the census of all female adolescents who had received a LARC from the same clinic (n=107). This second sample was manually cross-checked to verify that these individuals had an office visit for this reason to avoid wrongful survey assignment (all had in fact received a LARC insertion).

### Survey Implementation

Using the patient portal, clinic physicians sent automated email messages to potential participants. Once logged in to the portal, adolescents received an invitation and a link to the designated survey and consent form that assured anonymity. Given that adolescents may not check email frequently, we sent 4 reminders between August and October 2016. While the survey was anonymous, all respondents were offered a $5 email gift card for survey completion with each email and in the informed consent document, whereby they provided an email address on a different website. For the secondary aim of examining if adolescents might use portals for survey research, we tracked the rates of click-through messages read and surveys opened to see how many adolescents accessed and opened messages for participation but did not complete the survey.

## Results

Table 1 summarizes the similar mean age and usage of possible participants within each group. Only 3 participants fully completed either survey, making it impossible to form inferences about their opinions. However, the process for performing research on adolescents remains possible as evidenced by the number of participants who read at least 1 email message (29/107 [27.1%] of LARC recipients and 39/150 [26.0%] of controls, Table 2). Additionally, of those who opened the messages, almost half of the LARC recipients (10/107, 9.3%) read through the entire survey, while less than half (6/150, 4.0%) did of those who received the survey asking about health and health care preferences.

**Table 1:** Characteristics of potential survey participants.

Characteristics^a^		Long-acting reversible contraception recipients (n=107)	Age-matched controls without long-acting reversible contraception (n=150)
Female, n (%)		107 (100)	150 (100)
**Age, years, mean (range)**		19.0 (16-24)	18.6 (14-25)
	Younger than 18 years, n (%)	23 (21.5)	53 (35.3)
	With self-activated online portal, n (%)	89 (83.1)	107 (71.3)
**Age of those with activated online portal, mean**		19.0	18.6
	Younger than 18 years with self-activated online portal, n (%)	21 (20.0)	51 (33.7)
**Age of those without self-activated online portal, mean**		18.9	18.8
	Younger than 18 years, no self-activated online portal, n (%)	31 (29.4)	60 (40.0)

^a^None of these comparisons is statistically significant.

**Table 2:** Online portal activity of potential survey participants.

Characteristics^a^		Long-acting reversible contraception recipients (n=107)	Age-matched controls without long-acting reversible contraception (n=150)
Activated online portal (patient-dependent step), n (%)		89 (83.1)	107 (71.3)
Number of messages sent, n		525	600
Number who received up to 4 messages, n (%)		89 (83.1)	104 (97.2)
Number who read at least 1 message, n (%)		29 (27.1)	39 (26.0)
Number who logged into the online portal, n (%)		22 (20.5)	27 (18.0)
**Number of days until login after first message sent, n**			
	0-7 days	7	8
	8-15 days	7	4
	>16 days	8	15
Number who opened and reviewed survey, n (%)		10 (9.3)	6 (4.0)
Number who completed the survey, n (%)		2 (1.9)	1 (0.7)

^a^None of these comparisons is statistically significant.

## Discussion

### Principal Findings

This study demonstrates a first step toward using an adolescent patient portal, originally designed for confidentiality, as a mechanism for promoting survey research directly to adolescents. We are encouraged that so many of the adolescents read the messages sent to them and were at least willing to open the patient portal to access the survey even if we cannot form meaningful conclusions about the adolescent and youth points of view. Click-through rates revealed that approximately a quarter of adolescents did in fact read the message, and almost half of those read the survey; they simply did not want to complete the survey provided. It is possible that completion rates may have been higher if we had used a shorter survey or a topic that the adolescents found more engaging. This study confirms that response rates in adolescents are difficult to predict and are likely to be even lower when covering sensitive topics [10]. While we cannot comment on this population’s health literacy or opinions on LARCs, we can confirm that adolescents responded to email solicitation for patient portal participation, leaving opportunities open for future research. We believe such research will finally bridge the gap between needing to understand adolescent opinions and maintaining appropriate confidentiality.

### Limitations

There are several limitations that warrant discussion. First, the number of adolescents who have active portal accounts and opened messages in this sample may be higher than the general population due to constant promotion of the adolescent portal in this clinical setting [4]. Future studies would need to take portal activation rates into consideration. Conversely, given the high activation rate in this population, these adolescents may have had concerns that their provider would learn information about them that they did not want them to have. Second, having a larger and more diverse sample may yield meaningful completion rates; we were limited by the number of female youths who had received LARCs. Third, the topic of the survey may not have been engaging enough for the adolescents, and a different survey topic may achieve greater response and completion. Finally, the email solicitation or series of clicks that adolescents had to perform to reach the survey may have been inhibitive. Some electronic health records have internal survey-building capacity or text-based options and should be encouraged as future avenues for research.

### Conclusion

Online portals offer an important potential as a medium for adolescent research, but topic selection and methods of engagement need to be refined. The electronic health record system at this health institution will soon begin offering texts from the portal and internal surveys, potentially increasing adolescent response rates. Future research should ask adolescents, through interviews or focus groups, especially those who were identified for participation for this study, what mechanisms and content they prefer when discussing confidential topics and what possible barriers and facilitators they perceive.

## Abbreviations

LARC: long-acting reversible contraceptive
